# Resurgence of *Mycoplasma pneumoniae* infections in children: emerging challenges and opportunities

**DOI:** 10.1097/QCO.0000000000001126

**Published:** 2025-08-06

**Authors:** Ruben C.A. de Groot, Bianca M.M. Streng, Louis J. Bont, Patrick M. Meyer Sauteur, Annemarie M.C. van Rossum

**Affiliations:** a Department of Pediatrics, Erasmus MC University Medical Center Rotterdam – Sophia Children's Hospital, Rotterdam; b Department of Pediatric Infectious Diseases and Immunology, Wilhelmina Children's Hospital, University Medical Center Utrecht, Utrecht, The Netherlands; c Division of Infectious Diseases and Hospital Epidemiology, Children's Research Center, University Children's Hospital Zurich, University of Zurich, Zurich, Switzerland; d Department of Pediatrics, subdivision of Pediatric Infectious Diseases and Immunology, Erasmus MC University Medical Center Rotterdam – Sophia Children's Hospital, Rotterdam, The Netherlands

**Keywords:** children, epidemiology, macrolide resistance, *Mycoplasma pneumoniae*, respiratory tract infections

## Abstract

**Purpose of review:**

To summarize recent advances in *Mycoplasma pneumoniae* epidemiology, pathophysiology, diagnostics, and treatment, since the 2023–2024 global resurgence of *M. pneumoniae* following the COVID-19 pandemic has provided new insights.

**Recent findings:**

The remarkably prolonged reduction of *M. pneumoniae* infections during COVID-19-related nonpharmaceutical interventions has shed new light on *M. pneumoniae* transmission, both on an individual and a global level. *M. pneumoniae* epidemiology showed striking differences in comparison with other respiratory pathogens, including RSV and pneumococcus. We discuss the possible mechanisms behind the delayed resurgence, including waning immunity and the persistence of *M. pneumoniae* reservoirs. There have been contrasting reports on disease severity with notable differences in severity between children and adults, with young adults showing marked vulnerability. The inability of *M. pneumoniae* diagnostic tests to differentiate between infection and carriage poses a continuing challenge: in daily clinical practice as well as in the interpretation of study results. Furthermore, several studies report safety and utility for tetracyclines and fluoroquinolones as treatment alternatives to macrolide antibiotics.

**Summary:**

The global resurgence of *M. pneumoniae* following COVID-19 pandemic restrictions has provided a unique opportunity to study its epidemiology and pathophysiology, which has advanced our understanding of *M. pneumoniae* infections in children.

## INTRODUCTION

*Mycoplasma pneumoniae* is a common cause of respiratory tract infections in children. *M. pneumoniae* is a member of the bacterial class of Mollicutes and can be divided into two subtypes. Although *M. pneumoniae* is a well known cause of atypical pneumonia in children, the incidence of *M. pneumoniae* infections in adults is less clear. Apart from pulmonary disease, which is the main burden of *M. pneumoniae* infection, *M. pneumoniae* can lead to extrapulmonary manifestations, most notably mucocutaneus and neurological disease [1]. *M. pneumoniae* infections occur in a distinct pattern of upsurges every 1–5 years [2–5]. Crowding leads to increased rates of transmission, for instance within families, schools, army barracks or residential healthcare institutions [6–8]. Diagnosing *M. pneumoniae* infections can be challenging as the interpretation of positive PCR results for *M. pneumoniae* in upper respiratory tract samples is complicated by the presence of upper respiratory tract carriage. Upper respiratory tract carriage can occur in children, in particular during *M. pneumoniae* upsurges, when it can reach a prevalence up to 50% [9]. Furthermore, *M. pneumoniae* serology can be positive due to previous *M. pneumoniae* infection, hindering decision-making based on test results of acute phase single-sample serology. Due to the absence of a cell wall, options for antibiotic treatment in *M. pneumoniae* infections are limited to macrolides, tetracyclines and/or fluoroquinolones. Macrolides are first choice for antibiotic treatment in children, given the potential side effects of tetracyclines and fluoroquinolones in young children. Macrolide resistance is a growing concern, in particularly in East Asia, where the prevalence of macrolide resistance amongst *M. pneumoniae* isolates ranges up to 90% [10].

Many key features of *M. pneumoniae* epidemiology, diagnostics and treatment are still unknown. In this review, we will describe recent advances in our understanding of *M. pneumoniae* pathophysiology and disease characteristics in children. We will especially focus on the 2023–2024 global resurgence of *M. pneumoniae,* as this was the first *M. pneumoniae* outbreak after the COVID-19 epidemic. The impact on *M. pneumoniae* epidemiology by social distancing and other nonpharmaceutical interventions (NPIs) implemented because of the COVID-19 epidemic, has provided new insights. 

**Box 1:**
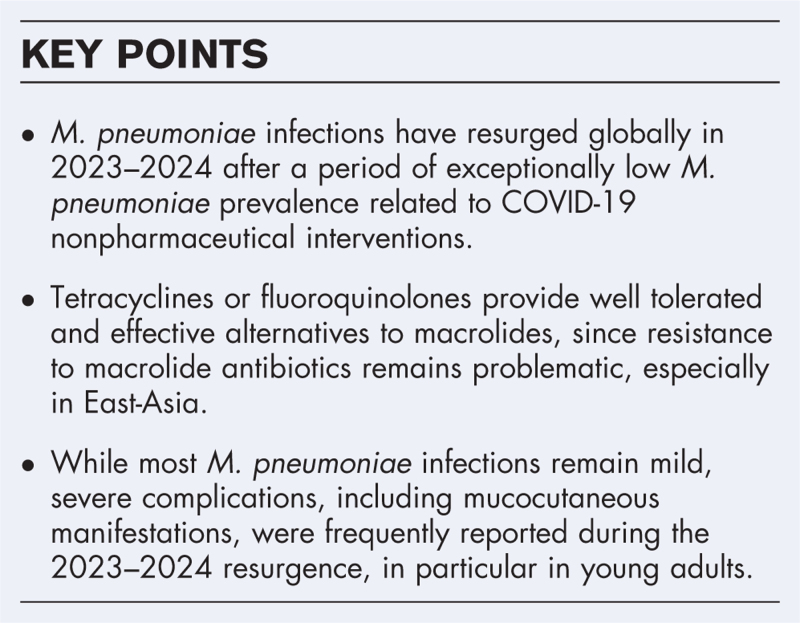
no caption available

## RESURGENCE OF *M. PNEUMONIAE* AFTER THE COVID-19 PANDEMIC AND LIFTING OF NON-PHARMACEUTICAL INTERVENTIONS

During social distancing and other NPIs implemented during the COVID-19 epidemic the global incidence of *M. pneumoniae* infections came to a sustained low level [11,12^▪^]. The second half of 2023, the fourth year after the initial implementation of NPIs against COVID-19, saw the re-emergence of *M. pneumoniae* with outbreaks of pneumonia amongst children and adults, and a steep rise in *M. pneumoniae* infection incidence [13,14^▪^,15^▪^]. Although epidemiological patterns previously varied per country, the last upsurge was noted in 2019. The 2023–2024 resurgence at first glance appeared to be more severe in numbers of *M. pneumoniae* infections and disease severity compared to previous ones. This global outbreak has been documented in more detail in publications from Australia [16], England [17], The Netherlands [18,19^▪^], Denmark [20], Switzerland [21], Italy [22], France [23], Spain [24,25], the USA [26–29], Colombia [30] and China [31–33] and lasted until 2024 (Table 1). This resurgence was observed long after NPIs were lifted [34]. What particular aspects within this miscellaneous bundle of NPIs, that differed between countries, most contributed to the decline in *M. pneumoniae* prevalence in the population remains unclear.

**Table 1:** Details of regional studies describing the 2023–2024 resurgence

Publication	Country	Cohort type	Study period
Graham *et al.* [16]	Australia	Reported test results in regional laboratory and a children's hospital	2016–2024
Todkill *et al.* [17]	United Kingdom	National respiratory surveillance program	2019–2024
Bolluyt *et al.* [18]	The Netherlands	Reported test results in regional laboratory and National respiratory surveillance program	2016–2023
Smit *et al.* [19^▪^]	The Netherlands	National respiratory surveillance program	2023–2024
Nordholm *et al.* [20]	Denmark	National surveillance program and nation-wide patient registry	2015–2023
Bouras *et al.* [21]	Switzerland	Reported test results in regional laboratory	2023–2024
Novazzi *et al.* [22]	Italy	Reported test results in regional laboratory	2023–2024
Edouard *et al.* [23]	France	Reported test results in a university hospital	2014–2024
Urbieta *et al.* [24]	Spain	Reported test results in a university hospital	2018–2023
Trobajo-Sanmartin *et al.* [25]	Spain	Regional healthcare system	2017–2024
Upadhyay *et al.* [26]	United States of America	Reported test results in a nation-wide laboratory service	2021–2023
Edens *et al.* [27]	United States of America	National respiratory surveillance program	2018–2023
Raghuram *et al.* [28]	United States of America	Nation-wide healthcare information system	2017–2024
Danner *et al.* [29]	United States of America	Reported test results in regional healthcare system	2020–2024
Gutierrez-Tobar *et al.* [30]	Colombia	Reported test results in several hospitals	2020–2023
Chen *et al.* [31]	China	Reported test results in several hospitals	2021–2023
Gong *et al.* [32]	China	Regional respiratory surveillance program	2023
Xu *et al.* [33]	China	Reported test results in several hospitals	2016–2023

## POST-PANDEMIC RESURGENCE OF OTHER RESPIRATORY PATHOGENS: IMPLICATIONS FOR *M. PNEUMONIAE*

The re-emergence of *M. pneumoniae* was delayed in relation to lifting of NPIs, also when compared to other respiratory tract pathogens, both viruses and bacteria. A respiratory syncytial virus (RSV) epidemic was observed within a half year of lifting the most stringent physical distancing measures in Western Australia, even out of the regular RSV season, namely during the Southern Hemisphere summer [35]. A similar pattern in RSV epidemiology was observed in the Northern Hemisphere [36,37].

Similarly rapid re-emergence after lifting of NPIs was observed for invasive bacterial infections including pneumonia, for instance by pneumococcus and Group A streptococcus [38–40]. There is no definitive explanation for the delayed *M. pneumoniae* resurgence compared to other respiratory tract pathogens, but there are several hypotheses. One hypothesis would be that viruses make an essential contribution to the pathogenesis of *M. pneumoniae* lower respiratory tract infection, for instance by leading to bacterial translocation from the upper to the lower respiratory tract, since this has been documented in detail for *Streptococcus pneumoniae* (pneumococcus) and *Streptococcus pyogenes*[39–42]. Furthermore, analogous to other bacterial causes of lower respiratory tract infection, upper respiratory tract viral infection could contribute to *M. pneumoniae* transmission in the population, explaining the delayed resurgence of *M. pneumoniae* when compared to respiratory viral infections after lifting of NPIs. Indeed, co-detection of *M. pneumoniae* with respiratory tract viruses is common [9]. However, dependency of *M. pneumoniae* on respiratory tract viruses for development of lower respiratory tract infection might explain why the *M. pneumoniae* resurgence is delayed compared to viruses, but not why it lagged behind the re-emergence of other bacterial respiratory tract pathogens. The hypothesis that delayed *M. pneumoniae* re-emergence could be explained by interactions with respiratory tract viruses thus seems unlikely. Another hypothesis could be related to differences in upper respiratory tract carriage. Previously, the introduction of NPIs had led to a swift and pronounced decrease in the incidence of viral and bacterial lower respiratory tract infections [40]. Interestingly, NPIs had no effect on the prevalence of pneumococcal carriage [43]. However, at this point we lack detailed data on *M. pneumoniae* carriage during the NPI and resurgence period. If indeed NPIs had an impact on the prevalence of *M. pneumoniae* carriage, which did not affect pneumococcal carriage, this could explain the discrepancy in the timing of resurgence between *M. pneumoniae* and *S. pneumoniae.*

## CONTRIBUTION OF *M. PNEUMONIAE* INTRINSIC FACTORS TO THE *M. PNEUMONIAE* RESURGENCE

The simultaneous reappearance of *M. pneumoniae* globally makes it unlikely that the resurgence resulted from the epidemic spread of *M. pneumoniae* from one particular geographical location. This suggests that some individuals still carried *M. pneumoniae* when social distancing measures were in place. These individuals could have been local reservoirs of the multiple observed simultaneous outbreaks. Notably, *M. pneumoniae* carriage has not been reported when NPIs were in place. Furthermore, it is surprising that the timing of the resurgence was so similar across the world, suggesting *M. pneumoniae* displays similar population transmission kinetics despite very different settings in terms of population density, meteorological factors, mobility, etc. Multiple origins of the *M. pneumoniae* resurgence would also suggest that several different *M. pneumoniae* subtypes are still present. Interestingly, data from previous upsurges as well as the 2023–2024 resurgence have shown that during a single outbreak, one *M. pneumoniae* subtype can be dominant, but more than one subtype are always present [19^▪^,^44^–^47^]. Indeed, metagenomic data from China suggests that during COVID-19-related NPIs, the same *M. pneumoniae* strains were still circulating [48^▪^]. An important feature of *M. pneumoniae,* likely to be essential for transmission trough the population, is *M. pneumoniae'*s slow generation time (±6 h). This results in a longer incubation period, and relatively low basic reproduction number (R_0_), which is likely a key contributor to the delayed resurgence compared to other bacterial and viral pathogens. Although data on *M. pneumoniae* incubation time are limited, a seminal study from 1966 describes a median incubation time of 23 days, mostly ranging from 2–4 weeks [49]. A 3-week incubation period showed an excellent fit in a global modelling study of the 2023–2024 resurgence [15^▪^].

## EFFECTS OF WANING IMMUNITY OF THE *M. PNEUMONIAE* RESURGENCE

A modelling study of previous *M. pneumoniae* outbreaks and the 2023–2024 resurgence suggested that the delayed reemergence can partly be explained by waning immunity to *M. pneumoniae* in the population [50]. Waning immunity has been proposed as an important mechanism in the changed dynamics of post-COVID-19 outbreaks of RSV, severe Group A streptococcus and parvovirus B19 [35,39,51]. Indeed, the role of antibody responses in *M. pneumoniae* carriage and infection has been well described. Upper respiratory tract *M. pneumoniae-*specific IgA blocks acquisition of *M. pneumoniae*[52,53] and hypogammaglobulinemia leads to more severe *M. pneumoniae* infection [54–56]. However, the unusual aspect of the *M. pneumoniae* epidemiology related to COVID-19 was not that the resurgence took particularly long, since 1–5 year cycles are common for *M. pneumoniae.* Waning immunity due to the duration of the inter-epidemic period should therefore not be more pronounced when compared to previous *M. pneumoniae* outbreaks. However, in contrast to previous inter-epidemic periods, levels of endemic *M. pneumoniae* circulation during COVID-19 NPIs was substantially lower. Low levels of endemic circulation could provide some population level immunity, which could have been absent during the current resurgence. In contrast with this hypothesis, a global *M. pneumoniae* epidemiology study found no relationship between declining levels of *M. pneumoniae*-specific antibodies and the *M. pneumoniae* resurgences [15^▪^]. In conclusion, it seems unlikely that waning immunity contributed to the delayed resurgence of *M. pneumoniae*, however at this point we lack data on a potential contribution of waning immunity to disease severity during the 2023–2024 *M. pneumoniae* resurgence.

## DISEASE SEVERITY IN CHILDREN DURING THE 2023–2024 RESURGENCE

A multicentre retrospective study in Spain found that *M. pneumoniae* infections during the 2023–2024 resurgence were more severe, with children requiring PICU admission in 14% of cases, with 44% of those admitted to the PICU requiring noninvasive or mechanical ventilation. These percentages are higher than found in studies on previous outbreaks, but importantly the study itself lacked a control group [57]. Similarly, a study in China showed a prolonged hospital stay, but could not exclude confounding factors, such as differences in age and season [58]. In contrast, studies in Portugal and China, with pre-COVID-19 cases as historical controls, found that *M. pneumoniae* infections in the 2023/2024 outbreak were not more severe. [59^▪▪^,60^▪^]. In line with these studies, a well controlled nation-wide study in Denmark based on health-care data showed that *M. pneumoniae* infections were not more severe compared to infections during previous *M. pneumoniae* upsurges [61^▪▪^]. This study from Denmark did report more mucocutaneous manifestations due to *M. pneumoniae*, that is, *M. pneumoniae*-induced rash and mucositis (MIRM) or *M. pneumoniae*-triggered reactive infectious mucocutaneous eruption (RIME) [61^▪▪^]. An outbreak of these mucocutaneous manifestations after *M. pneumoniae* infections was also reported in Australia during the 2023–2024 resurgence [62]. A study from the USA also reported high frequency of mucocutaneous eruptions, with 19.8% of patients with *M. pneumoniae* infection presenting with MIRM [29]. Although high numbers of severe *M. pneumoniae* infections are reported, it is difficult to determine if this is due to a higher percentage of total *M. pneumoniae* infections resulting in severe disease compared to previous epidemics. Alternatively, the high number of severe *M. pneumoniae* infections could reflect a high number of total *M. pneumoniae* infection. Disease severity during this epidemic is thus difficult to determine, since it has proven difficult to ascertain the exact total number of *M. pneumoniae* infections in the population including mild cases that were not medically attended. Taken together, these different studies report mixed results on disease severity, but until now there is no strong evidence that *M. pneumoniae* infections generally were more severe compared to previous epidemics.

## LESSONS LEARNED FROM *MYCOPLASMA PNEUMONIAE* INFECTIONS IN ADULTS DURING THE 2023–2024 RESURGENCE

In line with the infections in children, studies in adults show differences in reports of disease severity [63]. When compared to children, adults did generally present with milder disease and lower fever, but there have been several reports of severe *M. pneumoniae* infections during this outbreak with increased numbers of adults admitted to the intensive care [64–67]. Strikingly, severe *M. pneumoniae* infections do not occur more frequently in elderly patients, in fact young adults without comorbidities were more frequently affected [68,69]. This shows an interesting analogy with children, as comorbidities in children do not increase risk of severe *M. pneumoniae* infection [70]. Since young adults seem to be at increased risk for *M. pneumoniae* infection, this raises the question if this is the result of household transmission of *M. pneumoniae*, where these young adults are parents infected by their children. Family-clustered *M. pneumoniae* infections have also been described during the current resurgence [71]. In line with this, the age distribution in adults with *M. pneumoniae* infection is skewed towards young adults, which suggests a continuum with adolescents, with adult patients forming the tail of a gaussian curve [72]. However, some countries have reported a bimodal age distribution in adults, which is suggestive of family transmission [2]. Overall, transmission outside families with children seems to play an important role. *M. pneumoniae* transmission in schools could be one of those alternative explanations, especially since school closures were a prominent feature of NPIs. Indeed, for RSV reopening of schools was closely associated with viral re-emergence [73,74]. This would of course not explain the abundant reports of *M. pneumoniae* infections in adults, who seemed to be more affected during this resurgence. However, broad uptake of respiratory pathogen testing using PCR panels including PCRs for *M. pneumoniae* could provide an alternative explanation, where (severe) *M. pneumoniae* infection in adults in the past has been frequently underrecognized and underreported.

## *M. PNEUMONIAE* DIAGNOSTICS

*M. pneumoniae* diagnostics available in clinics are based on direct bacterial detection or serology. For direct detection antigen-based assays or *M. pneumoniae* PCR are used, usually on upper respiratory tract samples such as throat swabs, which will also be positive during *M. pneumoniae* carriage [9,75]. Serology includes acute phase *M. pneumoniae-*specific IgM, which if positive can mean a current infection but a previous infection as well, since *M. pneumoniae-*specific IgM remains positive for months. The reference standard remains a fourfold increase in *M. pneumoniae-*specific IgG. Since this requires a convalescent sample obtained 2–3 weeks after the acute phase sample, seroconversion does not provide a timely diagnosis of *M. pneumoniae* infection. Attributing cases of respiratory tract infections to *M. pneumoniae* thus continues to be complicated by the lack of a clinically robust reference standard. All reports during this resurgence are based on diagnostic tests that do not discriminate between *M. pneumoniae* infection or carriage. However, it seems likely that on a population level, the prevalence of upper respiratory tract carriage of *M. pneumoniae* has a similar kinetic in time when compared to *M. pneumoniae* infections. A study in children with recurrent respiratory tract infections found that *M. pneumoniae* was carried by 68% of children [76], and associated with a less diverse upper respiratory tract microbiome composition. Because of a lack of robust diagnostic tests, it remains unclear what the role is of *M. pneumoniae* in individual children with recurrent respiratory tract infections. However, some progress has been made in *M. pneumoniae* diagnostic testing in recent years. Levels of the biomarker TNF-related apoptosis-induced ligand (TRAIL) in peripheral blood were found to be elevated in children with *M. pneumoniae* infection when compared to children with a non-*M. pneumoniae* respiratory tract infection. While this may sound promising, the added diagnostic value of a combination of TRAIL, Interferon gamma-induced protein 10 (IP-10) and C-reactive protein (CRP) to an assessment based on clinical characteristics was limited, since the addition of TRAIL, IP-10 and CRP to clinical characteristics did not improve discriminatory accuracy for *M. pneumoniae* infection [77,78]. Another study investigated the use of host-response transcriptomics to identify *M. pneumoniae* with good performance (area under the curve of 0.84–0.95) [79], although it was unclear how this study definitively ascribed pneumonia cases to *M. pneumoniae*. The ultimate diagnostic test with superior sensitivity and specificity, which ideally would also provide point-of-care results, has not yet been identified. However, the likelihood of *M. pneumoniae* infection can be estimated using clinical characteristics, such as age more than 5 years, prodromal fever and respiratory symptoms more than 6 days, and CRP or PCT levels that are normal or only slightly elevated, etc. These clinical characteristics have been validated in a patient cohort were *M. pneumoniae* infection was ascertained using a *M. pneumoniae-*specific IgM antibody-secreting cell (ASC) enzyme-linked immunospot (ELISpot) assay, which can discriminate between *M. pneumoniae* carriage and infection [70]. Until there is a diagnostic test that can differentiate carriage from infection, focus should shift from diagnostic studies towards utility studies. Utility studies validate certain diagnostics test that can identify groups of patients likely to have *M. pneumoniae* infection, thereby identifying patient groups that would have clinical benefit from antibiotics with in-vitro effectiveness against *M. pneumoniae*.

## CHALLENGES FOR *M. PNEUMONIAE* TREATMENT: MACROLIDE RESISTANCE

Notably, there are no trials demonstrating clinical efficacy of macrolides in children with *M. pneumoniae* infection, which is related to the diagnostic issues described above. However, promising studies are underway, such as a trial aiming to show macrolide efficacy in patients who screened positive for *M. pneumoniae* using a *M. pneumoniae*-specific IgM point-of-care assay and will retrospectively be verified by specific PCR and IgM ASC ELISpot [80]. Diagnostic accuracy in these types of trials ideally will be monitored in a research setting using *M. pneumoniae-*specific IgG seroconversion or IgM ASC ELISpot [75]. With a diagnostic test that at least substantially enriches for patients with *M. pneumoniae* infection studies could demonstrate macrolide efficacy. In patients with probable or proven *M. pneumoniae* infection, macrolide antibiotics are first-line treatment, since *M. pneumoniae* is intrinsically resistant to beta lactam antibiotics because of its lack of a cell wall. Macrolide resistance amongst *M. pneumoniae* isolates remains high, in particular in East-Asia. Several regional studies demonstrate a prevalence of macrolide resistance up to 90% in China [59^▪▪^,^81^,^82^]. In contrast to reports from Asia, recent studies in Germany and the USA continue to find low levels of macrolide resistance [83,84]. Mutation analyses show that A2063G is the predominant cause of macrolide resistance [85], albeit that macrolide resistance testing is biased since it is almost only performed on genotype level. Culturing *M. pneumoniae,* and subsequent phenotypical antibiotic resistance testing, is usually not achievable and culture-based studies are thus lacking. The predominance of A2063G mutation can also be explained by the fact that this single point mutation is sufficient for macrolide resistance and can easily arise, particularly in the context of low macrolide concentration, that is just above MIC, which readily occurs in macrolides with long half-life such as azithromycin [86]. At this point there is little evidence to suggest macrolide resistance has changed during the resurgence.

## ROLE FOR TETRACYCLINES AND QUINOLONES IN TREATMENT OF *M. PNEUMONIAE* INFECTION

The persistent problem of macrolide resistance has spurred the study of other classes of antibiotics, particularly tetracyclines. Previously, tetracyclines have been avoided in children because of safety concerns. Enamel discoloration is frequently seen after tetracycline use [87], however it has long been recognized to be uncommon after doxycycline treatment [88]. Recent experiences after the reintroduction of broader use of doxycycline for Q fever and Rickettsiosis in particular, have confirmed that enamel discoloration after doxycycline treatment is rare, even in children under 8 years old [89,90]. Over the last couple of years, several studies have evaluated the use of tetracyclines and quinolones for *M. pneumoniae* infection. Although prone to bias due to the retrospective nature of these studies, these reports indicate that tetracyclines and quinolones could be effective alternatives to macrolide antibiotics [91^▪^,92^▪^].

## IMMUNOMODULATORY TREATMENT

Given high rates of macrolide resistance and the occurrence of macrolide refractory *M. pneumoniae* infections, immunomodulatory treatment is a relevant treatment option. Indeed, immunopathology is known to enhance disease, especially in severe *M. pneumoniae* infections [93]. There is sufficient evidence that adding steroids in refractory *M. pneumoniae* pulmonary infections is beneficial [94,95], although there is limited evidence for the exact dose and duration. During the 2023–2024 resurgence, there are no new studies in children that provide more evidence for immunosuppressive treatment during severe *M. pneumoniae* infection, however a study in adults found that adjunctive corticosteroid treatment shortened duration of fever [96^▪^].

## CONCLUSION

The 2023–2024 resurgence of *M. pneumoniae* followed the lifting of COVID-19 NPIs and has provided valuable insights in *M. pneumoniae* epidemiology. Studies on disease severity yielded mixed results, without showing strong evidence that *M. pneumoniae* infections generally were more severe compared to previous upsurges. The delayed resurgence compared to other respiratory pathogens may be due to a combination of factors such as viral interactions, bacterial carriage dynamics, and most likely to intrinsic characteristics of *M. pneumoniae*, including its slow generation time. Challenges in diagnosing *M. pneumoniae* persist due to carriage and limitations in current diagnostic tools. Treatment remains complicated by macrolide resistance, particularly in East Asia, though tetracyclines and quinolones are emerging as useful alternatives for children.

Many challenges remain, in particular the urgent need for improved diagnostic tests that can identify patients with *M. pneumoniae* infection that would benefit from antibiotic treatment and predict disease severity. Furthermore, more studies are needed to define the exact role of immunomodulatory treatment, and it in what subset of patients might benefit from these interventions. International collaboration during the 2023–2024 resurgence has proven to be pivotal in advancing our understanding of *M. pneumoniae* infection in children. To address the remaining challenges, the scientific community again needs to join efforts to answer these critical questions and improve the health of children with *M. pneumoniae* infection.

## Acknowledgements

*None.*

### Financial support and sponsorship

*None.*

### Conflicts of interest

*There are no conflicts of interest.*

## REFERENCES AND RECOMMENDED READING

Papers of particular interest, published within the annual period of review, have been highlighted as:

▪ of special interest
▪▪ of outstanding interest
